# Supplementary material for: From sequence to enzyme mechanism using multi-label machine learning
Source: BMC Bioinformatics. 2014 May 19;15:150. doi: 10.1186/1471-2105-15-150 (PMC4229970; doi:10.1186/1471-2105-15-150)
Supplement: Additional file 2 — Java code of ml2db. Additional file ml2db_code.tar.gz contains the Java source code to run the multi-label machine learning experiments and save the results to database. The code’s Javadoc is included. [file 1471-2105-15-150-S2.zip › additional file 2/ml2db/utils/doc/uk/ac/standrews/utils/main/stats/TruncatedPareto.html]

TruncatedPareto


JavaScript is disabled on your browser.


- Overview
- Package
- Class
- Use
- Tree
- Deprecated
- Index
- Help

- Prev Class
- Next Class

- Frames
- No Frames

- All Classes

- Summary:
- Nested |
- Field |
- Constr |
- Method

- Detail:
- Field |
- Constr |
- Method


uk.ac.standrews.utils.main.stats

## Class TruncatedPareto

- java.lang.Object
- - cern.colt.PersistentObject
  - - cern.jet.random.AbstractDistribution
    - - cern.jet.random.AbstractContinousDistribution
      - - cern.jet.random.Pareto
        - - uk.ac.standrews.utils.main.stats.TruncatedPareto

- All Implemented Interfaces:
  :   DoubleFunction, IntFunction, java.io.Serializable, java.lang.Cloneable

  Direct Known Subclasses:
  :   PseudoTruncatedPareto

  ---

    

  ```
  public class TruncatedPareto
  extends Pareto
  ```

  A utility class to extract from a truncated Pareto (power-law, long-tail)
  distribution. Numbers are extracted from a standard Pareto distribution. If
  the number extracted in above the truncation threshold, another extraction is
  performed. http://en.wikipedia.org/wiki/Pareto\_distribution

  Version:
  :   19 Jul 2008

  Author:
  :   Luna De Ferrari luna.deferrari-at-ed.ac.uk

  See Also:
  :   Serialized Form

- - ### Constructor Summary

    Constructors

    Constructor and Description

    `TruncatedPareto(double scale,
    double shape,
    int offset,
    int truncationThreshold)`
  - ### Method Summary

    Methods

    | Modifier and Type | Method and Description |
    | --- | --- |
    | `int` | `getOffset()` |
    | `Pareto` | `getPareto()` |
    | `java.util.TreeMap<java.lang.Integer,java.lang.Integer>` | `getParetoFrequencies(int numberOfExtractions)` Extracts numbers from a pareto distribution for the given number of times and records the freqencies with which the integers have been extracted |
    | `int` | `getTruncationThreshold()` |
    | `int` | `nextInt()` Returns a random number from the distribution; returns (int) Math.round(nextDouble()). |

    - ### Methods inherited from class cern.jet.random.Pareto

      `cdf, nextDouble, nextDouble, pdf, setState, staticNextDouble, toString`
    - ### Methods inherited from class cern.jet.random.AbstractDistribution

      `apply, apply, clone, makeDefaultGenerator`
    - ### Methods inherited from class java.lang.Object

      `equals, getClass, hashCode, notify, notifyAll, wait, wait, wait`

- - ### Constructor Detail


    - #### TruncatedPareto

      ```
      public TruncatedPareto(double scale,
                     double shape,
                     int offset,
                     int truncationThreshold)
      ```

      Parameters:
      :   `scale` - the distribution scale
      :   `shape` - the distribution shape
      :   `offset` - an offset (all extracted numbers will start from the offset)
      :   `truncationThreshold` - a truncation threshold (no extracted number will be above the
          threshold)
  - ### Method Detail


    - #### getOffset

      ```
      public int getOffset()
      ```


    - #### getPareto

      ```
      public Pareto getPareto()
      ```


    - #### getParetoFrequencies

      ```
      public java.util.TreeMap<java.lang.Integer,java.lang.Integer> getParetoFrequencies(int numberOfExtractions)
      ```

      Extracts numbers from a pareto distribution for the given number of times
      and records the freqencies with which the integers have been extracted

      Parameters:
      :   `numberOfExtractions` - the number of integer extractions from a a pareto distribution

      Returns:
      :   a map containing an integer as key and a frequency as value
          (frequency = number of times the integer has been extracted)


    - #### getTruncationThreshold

      ```
      public int getTruncationThreshold()
      ```


    - #### nextInt

      ```
      public int nextInt()
      ```

      **Description copied from class: `AbstractDistribution`**

      Returns a random number from the distribution; returns
      (int) Math.round(nextDouble()). Override this method if
      necessary.

      **Overrides:**
      :   `nextInt` in class `AbstractDistribution`


- Overview
- Package
- Class
- Use
- Tree
- Deprecated
- Index
- Help

- Prev Class
- Next Class

- Frames
- No Frames

- All Classes

- Summary:
- Nested |
- Field |
- Constr |
- Method

- Detail:
- Field |
- Constr |
- Method
